# Incidence and predictors of lost to follow-up among women under Option B+ PMTCT program in western Ethiopia: a retrospective follow-up study

**DOI:** 10.1186/s13104-019-4882-z

**Published:** 2020-01-07

**Authors:** Tadesse Tolossa, Getachew Mullu Kassa, Habtamu Chanie, Amanuel Abajobir, Diriba Mulisa

**Affiliations:** 1 Department of Public Health, Institute of Health Science, Wollega University, P.O.BOX: 395, Nekemte, Ethiopia; 2 Department of Midwifery, College of Health Science, Debre Markos University, Debre Markos, Ethiopia; 3 Maternal and Child Wellbeing Unit, African Population and Health Research Centre, Nairobi, Kenya; 4 Department of Nursing, Institute of Health Science, Wollega University, Nekemte, Ethiopia

**Keywords:** Lost, Option B+, Mother-to-child transmission, Ethiopia

## Abstract

**Objective:**

Although Ethiopia has been implementing Option B+ program, LTFU of women from the Option B+ program is one of the challenges that minimizes its implementation. Thus, this study assessed the incidence and predictors of LTFU among women under Option B+ PMTCT program in western Ethiopia. An institution-based retrospective follow-up study was conducted. A cox proportional hazards regression model was fitted to identify predictors of LTFU. A Hazard ratios with 95% confidence CI was computed and all predictors that were associated with the outcome variable at p-value ≤ 0.05 in the multivariable cox proportional hazards were declared as a significance predictor of the outcome.

**Results:**

A total of 330 women were followed for a mean follow up time of 16.9 (± 7.6) months. An overall incidence rate of LTFU was 9/1000 person-months. Women’s educational status, residence, HIV-disclosure status, the status of women at enrollment, previous history of HIV and ART adherence were significant predictors of LTFU. The incidence of LTFU from Option B+ PMTCT is lower as compared to evidence from sub-Saharan African and strengthening linkage and referral system between clinics as well as establishing appropriates tracing mechanisms would retain pregnant women in the program.

## Introduction

The number of new pediatric HIV infections was reduced from 270,000 in 2009 to 160,000 in 2017 worldwide, more than 90% of these children were infected through mother-to-child transmission (MTCT). That is, without intervention, the risk of MTCT is 15–30% during pregnancy and delivery and 5–20% during breastfeeding contributing for an overall transmission rate of 20–45% [1]. In western and Central Africa, the rate of MTCT of HIV was 20.2%, and around 180,000 children acquired HIV during delivery and breast-feeding in 2017 [2].

The World Health Organization (WHO) guideline on ART recommends three options to prevent MTCT transmission of HIV infection—Options A, B, and B+. The latest approach, Option B+ PMTCT program, emphasizes on the provision of universal, lifelong ART for all HIV-infected women regardless of CD4 count and WHO clinical staging [3]. Moreover, WHO developed guidelines recommending a ‘treat all’ approach, meaning all people diagnosed with HIV should be offered immediate treatment. This has increased the number of women of reproductive age who are receiving ART, regardless of whether they are pregnant or not [4].

Ethiopia has been implementing Option B+ program since 2013 as part of its national policy for preventing new HIV infections among children and to improve maternal survival [5]. Under Option B+, all HIV-infected pregnant women will receive universal ART and will continue the treatment for the rest of their live [6] and have an advantage of simplification of ART, protection against MTCT in future pregnancies, a continuing prevention benefit against sexual transmission to serodiscordant partners, avoiding “stop start-stop” approach of antiretroviral drugs and minimize the opportunity of LTFU [7]. Before the implementation of option B+ PMTCT program, Ethiopia is one of the top 20 countries, where one children infected with HIV out of three child born to women living with HIV [5]. The proportion of pregnant women living with HIV who received antiretroviral medicines for the prevention of mother-to-child transmission has increased from a baseline of 37% in 2009 to 77% in 2014 and increased to 80% in 2017 and estimated 1.4 million infectious among children age 0–14 years was decreased from 2010 to 2017 [8, 9]. This indicates a major success for the Option B+ strategy and how it enables pregnant women to get antiretroviral therapy.

In the meantime, retention of pregnant and lactating women on universal lifelong ART is one of the crucial success indicators of the Option B+ program. This is particularly important to achieve optimal maternal health and successful PMTCT program throughout all stages of PMTCT services. However, loss to follow-up (LTFU) from the PMTCT program creates a great challenge for the successful implementation of option B+ program particularly in sub-Saharan Africa (SSA) where the prevalence of HIV is high [2, 10].

Overall, different studies show that younger age, lack of education, stigma and discrimination, failure to disclose HIV-status, long distances to reach health facilities, long waiting times, poor adherence to treatment, high CD4 count and WHO clinical stage I, II were associated with LTFU [11–13].

Unfortunately, LTFU from Option B+ decreases women’s access to HIV care and treatment, which leads to the advanced stage of HIV, increases maternal HIV/AIDS-related morbidity and mortality, facilitates the vertical transmission of HIV to newborn and facilitates the development of drug resistance [10, 14]. Even though Option B+ program has been implemented for the last 6 years, there are no studies and/or documented reports on the incidence of LTFU and its predictors among pregnant and lactating women on lifelong ART.

Therefore, this study aimed to assess the incidence and predictors of LTFU among women under the Option B+ program at Nekemte Specialized Hospital in western Ethiopia.

## Main text

### Methods

This study was conducted at Nekemte Specialized Hospital among women enrolled in Option B+ PMTCT program from 12 June 2013 through to December 1, 2018. The provision of ART services was initiated in 2005 in the hospital and the Option B+ PMTCT program started in 12 June 2013. A retrospective follow-up study was employed. From June 12, 2013 to December 1, 2018, 412 women were started ART under Option B+ PMTCT program at Nekemte specialized hospital. Of the total women who started ART under Option B+ program, 19 patients’ cards were not available. Three hundred ninety-three patient cards were reviewed and 63 patient cards were excluded due to incompleteness of the data (date of HIV diagnosis, date of ART initiation, recent date and outcome not recorded). Finally, 330 patient cards with complete data were included in the final analysis.

### Study variables

#### Dependent variable

Incidence of LTFU from Option B+ PMTCT.

#### Operational definition

- Event: LTFU which is 3 months after the last documented visit under Option B+ PMTCT and not recorded as ‘dead’ or ‘transferred-out’ on patient PMTCT logbook or medical cards [11, 15, 16].
- Censored: a patient did not develop an event or LTFU (death, transferred-out, treatment completed, and receiving treatment when the study was ended).
- Survival time: the time in months from the beginning of treatment under the Option B+ PMTCT program to LTFU from the program.

#### Data collection, data management, and analyses

The data were collected by using a checklist, developed from FMoH PMTCT logbook, ART intake forms and medical cards of eligible participants. The outcome variable, LTFU, was confirmed by reviewing the medical or PMTCT registration logbook at the hospital, which was recorded by health professionals working in the PMTCT clinic. Two trained nurses working at the PMTCT clinic were recruited for data collection, and one trained Public Health practitioner supervised the overall data collection.

Epidata version 3.2 was used for data entry, and then the data were exported to STATA version 14 for cleaning and further analyses. Descriptive non-parametric survival analyses such as the life-table and Kaplan–Meier survival curves were used for estimating survival probability.

To identify predictors of LTFU, bivariable and multivariable Cox regression analysis was conducted. To select variables for multivariable cox analysis, bivariable cox regression at p-value ≤ 0.25 were used. Then full multivariable cox analysis was conducted by using a backward stepwise selection process including all the potential risk factors that had a p-value of ≤ 0.25 in bivariable Cox proportional hazard analysis. Then the final best model for multivariable proportional hazard analysis was selected by using Log likelihood ratio (LLH). Hazard ratios (HR) with 95% confidence intervals (CIs) were computed and statistical significance was declared when it is significant at a 5% level (p-value < 0.05).

### Results

#### Sociodemographic and maternal characteristics of study participants

During the study period, a total of 393 women were initiated ART under option B+ PMTCT program at Nekemte specialized hospital. Of them, 330 (84%) women’s chart was included in the analysis and 63 (16%) charts were excluded due to incompleteness of the data. The mean (± standard deviation (SD)) age of women was 26.6 ± 4.5 years. One hundred fifty (45.4%) women belonged to 25–29 years’ age group. The study revealed that 199 (60.0%) study participants were urban residents, 217 (65.8%) were married. (Table 1).

**Table 1:** Baseline sociodemographic and maternal characteristics of women under Option B+ PMTCT program at Nekemte Specialized Hospital, western Ethiopia, 2013–2019

Variables	Category	Survival status		Total
		LTFU	Censored	
		No (%)	No (%)	No (%)
Age	≤ 24	26 (51.0)	67 (24.0)	93 (28.2)
	25–29	17 (33.3)	133 (47.7)	150 (45.4)
	≥ 30	8 (15.7)	79 (28.3)	87 (26.4)
Residence	Rural	34 (66.7)	97 (34.8)	131 (39.7)
	Urban	17 (33.3)	182 (65.2)	199 (60.3)
Marital status	Never	13 (25.5)	41 (14.7)	54 (16.3)
	Married	30 (58.8)	187 (67.0)	217 (65.8)
	Widowed/divorced	8 (15.7)	51 (18.3)	59 (17.9)
Educational status	No education	22 (43.1)	63 (22.6)	85 (25.8)
	Primary	19 (37.3)	94 (33.7)	113 (34.2)
	Secondary and above	10 (19.6)	122 (43.7)	132(40.0)
Employment	Housewife	24 (47.1)	128 (45.9)	152(46.1)
	Gov’t employee	10 (19.6)	74 (26.5)	84 (25.5)
	Non gov’t employee	4 (7.8)	33 (11.8)	37 (11.2)
	Daily laborer	7 (13.7)	30 (10.8)	37 (11.2)
	Others	6 (11.8)	14 (5.0)	20 (6.0)
Partner HIV status	Positive	8 (15.6)	98 (35.1)	106 (32.1)
	Negative	24 (47.1)	113 (40.5)	137 (51.5)
	Unknown	19 (37.3)	68 (24.4)	87 (26.4)
Disclosure status	No	29 (56.9)	56 (20.1)	85 (25.8)
	Yes	22 (43.1)	223 (79.9)	245 (74.2)
Status of women on enrollment	Pregnant	39 (76.5)	249 (89.2)	288 (87.3)
	Lactating	12 (23.5)	30 (10.8)	42 (12.7)
Number of pregnancy	Single	28 (54.9)	86 (30.8)	114 (34.6)
	Multiple	23 (45.9)	193 (69.2)	216 (65.4)

LTFU—lost to follow up, others—students, not employed

#### Baseline clinical, laboratory and follow-up characteristics

Of the total observations, 271 (82.1%) of women were at WHO clinical stage I/II and 263 (79.70%) had a CD4 count greater than 351 cells/mm^3^ at baseline. The mean hemoglobin level at enrollment was 12.0 mg/dl. The predominant regimens initially prescribed were a combination therapy—TDF/3TC/EFV for 240 (72.7%) participants, followed by AZT/3TC/NVP for 51 (15.45%). During the follow-up period, 165 (50%) women took CPT at baseline and 306 (92.7%) were negative for TB screening and 160 (52.3%) took IPT.

#### Survival status of women

**Fig. 1:**
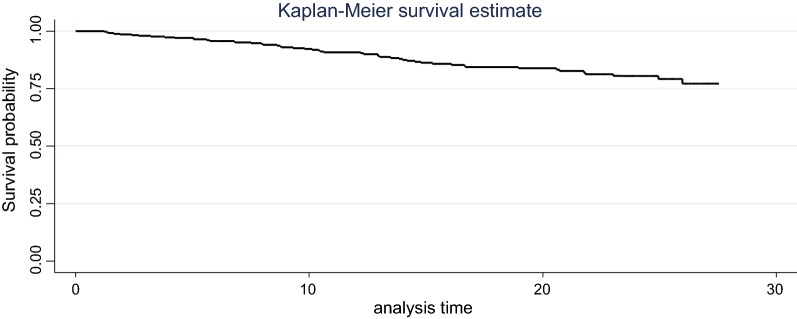
The Kaplan–Meier survival function estimates of HIV positive women under option B+ PMTCT program at Nekemte Specialized Hospital, western Ethiopia, 2013–2019

A total of 330 patients were followed for a mean time of 16.9 (SD ± 7.61) months. Two hundred seventy-nine (84.6%) observations were censored at the end of the study. During the follow-up time, a total of 5609.54 person-months’ time risk was observed with a minimum and maximum follow-up time of 1.2 and 27.5 months, respectively. Overall, 186 (56.4%) women completed the program and transferred to lifelong ART follow-up, 27 (8.2%) were transferred to other health institutions before completing the program (Fig. 1).

#### Incidence and time to LTFU

Of the total observations, 51 (15.4%) women were LTFU by the end of the study with an overall incidence rate of LTFU of 9 per 1000 (95% CI 6.8–11.9) person-months observations. The median time to LTFU from option B+ PMTCT program was 10.4 (95% CI 8.03–13.01) months. The highest incidence of LTFU was observed at 12th, 13th and 25th months of enrollment (18.1/1000, 18.9/1000 and 18.5/1000 person-months) observations, respectively.

#### Predictors of LTFU

Variables considered for multivariable cox regression analysis were those with a p-value < 0.25 in bivariable analysis and 16 variables were selected in the first step of model buildings. After running backward stepwise variables selection process, the first group was selected as the best model from LLH ratio table, which include full (9) variables (educational level, residence, disclosure status, partner HIV status, status of women on initiation of ART, previous history of HIV, hemoglobin level, level of ART adherence and baseline regimen) in multivariable analysis.

Six of the predictors (educational level, residence, disclosure status, status of women on initiation of ART, previous history of HIV and level of ART adherence) were found to have statistically significant association with outcome variable and found to be independent predictors of the incidence of LTFU during multivariable cox proportional regression analysis at 95% CI. The risk of LTFU among women who had no education was 4.01 times higher than women who had secondary and above education level (AHR = 4.01, 95% CI 1.84–8.76). Likewise, LTFU among women who were residing in the rural areas was 2.80 times higher than women who were residing in the urban areas (AHR = 2.80, 95% CI 1.54–5.08). The risk of LTFU among women who did not disclose their HIV status was 2.80 times higher than those women who disclosed their HIV status (AHR = 2.80, 95% CI 1.50–5.22). Moreover, initiation of ART program and previous history of HIV showed a statistically significant association with LTFU. That is, the risk of LTFU among women who were enrolled to the program after delivery was 4.10 times higher than women who initiated the program during pregnancy (AHR = 4.10, 95% CI 2.03–8.28) and its risk among women who were newly diagnosed for HIV on entry to PMTCT or no previous history of HIV was 3.04 times higher than women who had the previous history of HIV (AHR = 3.04, 95% CI 1.59–5.80). On last the follow-up, the risk of LTFU among women who had poor and fair level of adherence was 2.80 times higher than those women who had a good level of ART adherence (AHR = 2.80, 95% CI 1.29–6.07) (Table 2).

**Table 2:** Multivariable cox proportional analysis of incidence and predictors of LTFU among women under Option B+ PMTCT at Nekemte Specialized Hospital, western Ethiopia, 2013–2019

Variables	Category	Survival status		CHR	AHR	p-value
		LTFU	Censored			
		No (%)	No (%)			
Educational level	No education	22 (43.1)	63 (22.6)	3.95 (1.87–8.35)	4.01 (1.84–8.76)	< 0.001*
	Primary	19 (37.3)	94 (33.7)	2.23 (1.03–4.81)	1.63 (0.72–3.68)	0.232
	Secondary and above	10 (19.6)	122 (43.7)	1	1	
Residence	Rural	34 (66.7)	97 (34.8)	3.43 (1.91–6.14)	2.80 (1.54–5.08)	0.001^*^
	Urban	17 (33.3)	182 (65.2)	1	1	
Disclosure status	No	29 (56.9)	56 (20.1)	4.15 (2.38–7.24)	2.80 (1.50–5.22)	0.001*
	Yes	22 (43.1)	223 (79.9)	1	1	
Partner HIV status	Positive	8 (15.6)	98 (35.1)	1	1	
	Negative	24 (47.1)	113 (40.5)	2.25 (1.01–5.01)	2.27 (0.97–4.87)	0.059
	Unknown	19 (37.3)	68 (24.4)	3.26 (1.42–7.45)	1.90 (0.79–4.52)	0.146
Status of women on enrollment	Pregnant	39 (76.5)	249 (89.2)	1	1	
	Lactating	12 (23.5)	30 (10.8)	3.62 (1.84–7.09)	4.10 (2.03–8.28)	<0.001^*^
Previous history of HIV	Yes	20 (39.2)	201 (75.7)	1	1	
	No	31 (60.8)	78 (24.3)	3.84 (2.18–6.75)	3.04 (1.59–5.80)	0.001^*^
Hemoglobin	≤12.0	19 (37.3)	143 (51.3)	1	1	
	≥12.1	32 (62.7)	136 (48.7)	1.67 (0.95–2.96)	1.77 (0.97–3.25)	0.061
Recent adherence	Good	41 (80.4)	256 (91.8)	1	1	
	Fair/poor	10 (19.6)	23 (8.2)	2.40 (1.20–4.80)	2.80 (1.29–6.07)	0.009*
Baseline regimen	TDF/3TC/EFV	42 (82.4)	198 (80.0)	1		
	AZT/3TC/NVP	6 (11.8)	45 (16.1)	0.60 (0.25–1.43)	1.11 (0.43–2.85)	0.815
	AZT/3TC/EFV	3 (5.8)	36 (12.9)	0.40 (0.12–1.29)	0.44 (0.12–1.52)	0.198

*CHR* crude hazard ratio, *AHR* adjusted hazard

## Discussion

This study was conducted to determine the incidence of LTFU and its predictors among women under the Option B+ PMTCT program. The overall incidence rate of LTFU from the Option B+ PMTCT program (9/1000) was lower than the finding from south Wollo study in northeast Ethiopia (14.8/1000 person-months) [16]. This discrepancy might be due to the time differences when the studies were conducted. The previous study was conducted when the program was initiated for the first time in the country, which might have increased the rate of LTFU due to lack of resources allocated including trained human power, to implement the program effectively. The finding is also lower than studies conducted in different countries such as Myanmar, Uganda, South Africa, Malawi and Kenya [12, 14, 17–19]. These variations might be due to the differences in definitions of LTFU in different settings as indicated above.

The study also identified different predictors of LTFU from the program. For instance, consistent with previous studies from low and middle income-countries (LMICs), the risk of LTFU among women with no education was higher than women who had attended secondary and above education [12, 20, 21] where each additional year in school increases the likelihood of retaining to the program. Apparently, higher education levels contribute to better health literacy, self-care and greater access to information about the program. Moreover, consistent with findings Tanzania, Malawi and Uganda [15, 21, 22], the risk of LTFU among women residing in rural was higher as compared to women residing in urban, suggesting distance from home to hospitals, particularly in rural areas where women cannot easily get transport services due to physical barriers, forces women to walk long distances, may force them to miss appointments leading to poor adherence to ART.

HIV disclosure status significantly predicted LTFU in many low and middle-income countries [12, 13, 21, 23, 24]. This might be related to the stigma associated with the disease, fear of negative consequences from their partners and perceiving to preserve family stability.

The risk of LTFU among women who were enrolled in the program during breastfeeding was higher than women who were enrolled in the program during pregnancy [14, 25]. However, this finding is in contrast with studies conducted in Malawi in which the risk of LTFU from the Option B+ program was higher in pregnant women than women who enrolled to program after delivery [17, 26]. This discrepancy might be because of the difference on the date of ART initiation. In Malawi, most women start ART under Option B+ during pregnancy with the principle of “test and treat” approach. Hence, initiating ART on the same day with HIV diagnosis or under the “test and treat” approach increases the risk of LTFU from the program [27, 28].

The risk of LTFU among women who were newly diagnosed with HIV was consistently higher than women who were previously diagnosed with HIV [25, 29, 30].

In summary, the highest incidence of LTFU was observed in the last month of follow up which implies poor linkage and referral systems between PMTCT and ART clinics. Thus, it is better to strengthen the linkage and referral system between PMTCT and ART clinics. Further prospective cohort and qualitative study should be conducted to identify the relationship between different identified factors and LTFU.

## Limitation of the study

First, it is a retrospective review of records, which further limited due to unregistered outcomes and limited information regarding basic sociodemographic and clinical characteristics. Second, the true incidence of LTFU might be underestimated and/or overestimated due to the incomplete documentation of data and some of the participants classified as LTFU might have died or silently transferred to other health institutions.

## Data Availability

The datasets analyzed during the current study are available from the corresponding author upon reasonable request.

## Abbreviations

AHR: adjusted hazard ratio
ART: antiretro viral therapy
CI: confidence interval
CHR: crude hazard ratio
HIV: human immunodeficiency virus
LTFU: lost to follow up
MTCT: mother to child transmission
PMTCT: prevention of mother to child transmission
SSA: sub-Saharan Africa
WHO: World Health Organization
